# The Effect of Sodium Tanshinone IIA Sulfate and Simvastatin on Elevated Serum Levels of Inflammatory Markers in Patients with Coronary Heart Disease: A Study Protocol for a Randomized Controlled Trial

**DOI:** 10.1155/2013/756519

**Published:** 2013-08-04

**Authors:** Qinghua Shang, Hanjay Wang, Siming Li, Hao Xu

**Affiliations:** ^1^Graduate School, Beijing University of Chinese Medicine, Beijing 100029, China; ^2^Department of Cardiovascular Diseases, Xiyuan Hospital, China Academy of Chinese Medical Sciences, Beijing 100091, China; ^3^College of Physicians and Surgeons, Columbia University, New York, NY 10032, USA

## Abstract

*Background*. Coronary heart disease (CHD) due to atherosclerotic inflammation remains a significant threat to global health despite the success of the lipid-lowering, anti-inflammatory statins. Tanshinone IIA, a potent anti-inflammatory compound derived from Traditional Chinese Medicine (TCM), may be able to supplement statins by further reducing levels of circulating inflammatory markers correlated to cardiovascular risk. Here, we present the protocol of a randomized controlled trial (RCT) that will investigate the synergistic effect of sodium tanshinone IIA sulfate and simvastatin on reducing elevated inflammatory markers in patients with CHD. Participants: Seventy-two inpatients with confirmed CHD, elevated serum high-sensitivity C-reactive protein (Hs-CRP) level, and a TCM diagnosis of blood stasis syndrome will be enrolled and randomized 1 : 1 into the control or experimental group. *Intervention*. All subjects will receive a standard Western therapy including 20 mg simvastatin orally once per evening. Patients in the experimental group will additionally receive a daily 80 mg dose of sodium tanshinone IIA sulfate intravenously, diluted into 250 mL 0.9% NaCl solution. The treatment period will be 14 days. *Outcomes*. Primary outcome parameter: serum Hs-CRP level. Secondary outcome parameters: other circulating inflammatory markers (including IL-6, TNF**α**, VCAM-1, CD40, sCD40L, MCP-1, and MMP-9), improvement in symptoms of angina and blood stasis syndrome, and safety. This trial is registered with ChiCTR-TRC-12002361.

## 1. Background

Cardiovascular disease is the worldwide leading cause of death. Globally in 2008, over 17 million people died from cardiovascular diseases, representing 30% of total deaths around the world [1]. Among all cardiovascular diseases, coronary heart disease (CHD) is responsible for the greatest mortality, accounting for 7.3 million deaths worldwide in 2008 [1]. 

CHD involves narrowing of the arteries supplying oxygen to the heart, most often due to buildup of atherosclerotic plaque in the coronary vessels. The ultimate rupture of atherosclerotic plaque may lead to the onset of acute coronary syndrome (ACS), a medically emergent manifestation of CHD involving unstable angina or myocardial infarction. 

The formation of atherosclerotic plaque is an inflammatory response, usually associated with elevated levels of low-density lipoprotein cholesterol (LDL-C) in the blood [2]. To this end, statins have served as a notably successful pharmacologic intervention against CHD from the standpoint of Western medicine. One meta-analysis study found that the average statin regimen reduces LDL-C levels by 35%, leading to a 60% decrease in ischemic cardiac events such as those of ACS [3]. Remarkably, as demonstrated by the PRINCE study, statins also moderate the atherosclerotic inflammatory response by reducing levels of C-reactive protein (CRP) [4], a robust marker of systemic inflammation whose concentration in the blood strongly correlates with the patient's cardiovascular risk [5]. Nevertheless, a multicenter study involving over 4,000 ACS patients showed that 22.4% of patients receiving intensive statin therapy suffered a serious cardiovascular or cerebrovascular event within two years of initiating treatment [6], indicating that although statins may be one of Western medicine's most effective agents for CHD, the toll of this disease remains significantly high.

At East-West integrative medical centers in Asia, cardiovascular diseases may also be evaluated according to the principles of Traditional Chinese Medicine (TCM). Among patients with the Western diagnosis of CHD, the TCM diagnosis of blood stasis syndrome (BSS) is exceedingly common, and the treatment for these CHD-BSS patients via TCM frequently involves herbal therapies using the root of *Salvia miltiorrhiza* (*丹参*, danshen) [7].

The danshen root contains tanshinone IIA, an active biochemical compound that has been shown to possess a multitude of antiatherosclerotic properties [8]. Most noteworthy is the ability of tanshinone IIA to decrease the levels of numerous inflammatory mediators associated with the progression of atherosclerosis, such as CRP, interleukin-6 (IL-6), tumor necrosis factor alpha (TNF*α*), vascular cell adhesion molecule-1 (VCAM-1), CD40, monocyte chemotactic protein-1 (MCP-1), and matrix metalloproteinase-9 (MMP-9) [8, 9]. A recent clinical trial demonstrated that giving simvastatin in combination with intravenous sodium tanshinone IIA sulfate (STS), the most widely used clinical formulation of tanshinone IIA in China, significantly decreased the levels of CRP, cholesterol, and plaque buildup in patients with peripheral vascular disease [10]. Notably, the integrative therapy was safer and more effective in treating this form of atherosclerosis than simvastatin alone. Whether there is potential for synergy between STS and statins in treating CHD, however, remains unknown.

Here, we present the protocol for a randomized, controlled clinical study that applies East-West integrative medicine to the treatment of CHD. Using standard Western therapy involving simvastatin as a foundation, we aim to explore the potential of further dampening the atherosclerotic inflammatory reaction in CHD-BSS patients through the concomitant addition of STS, utilizing the compound's potent anti-inflammatory properties as a supplement to simvastatin. In addition, we also aim to assess the integrative therapy's safety and its efficacy in improving angina and BSS symptoms relative to the simvastatin regimen without STS. Overall, this study offers an entry point for understanding and verifying the clinical applications of STS-statin integrative therapy in treating patients with CHD. 

## 2. Methods/Design

### 2.1. Setting and Design

This trial is a monocentric, parallel-design, randomized, controlled, clinical pilot study that will be conducted at Xiyuan Hospital, and China Academy of Chinese Medical Sciences in Beijing, China. Subject recruitment is scheduled to begin in August 2012. This study will involve 72 patients with the diagnoses of CHD and BSS according to Western medicine and TCM, respectively. The 72 participants will be randomized 1 : 1 into a standard Western therapy (control) group and an East-West integrative therapy (experimental) group. For 14 days, all 72 patients will be treated with a standard Western therapy involving simvastatin, and patients in the experimental group will additionally receive intravenous STS. Data will be collected before initiating treatment, immediately after the 14-day treatment period and 30 days posttreatment. Serum CRP levels measured by high-sensitivity CRP (hsCRP) testing will serve as the primary outcome parameter, whereas the levels of other inflammatory markers, the improvement of angina and BSS symptoms, and safety will serve as secondary outcome parameters.

**Figure 1:**
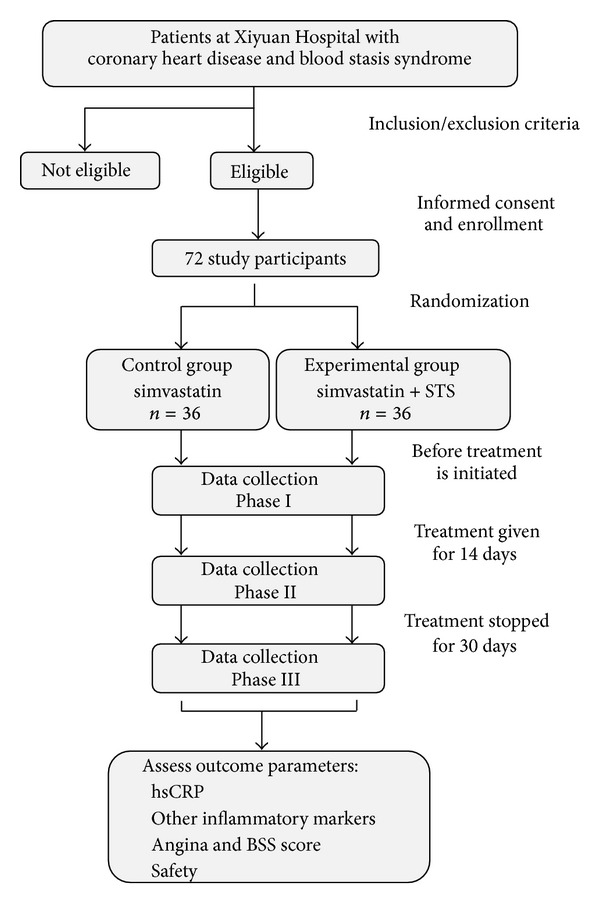
Study flowchart.

The study design is illustrated in Figure 1 and described in detail below according to the CONSORT 2010 statement [11].

### 2.2. Diagnostic Criteria

The diagnosis of CHD will be based on the standardized criteria established in “Nomenclature and criteria for diagnosis of ischemic heart disease,” a joint report published by the International Society and Federation of Cardiology and the World Health Organization [12]. In addition, following the current practice of CHD clinical research, we will require subjects to either have a previous history of myocardial infarction or have at least one coronary artery stenosis ≥50% confirmed by coronary angiography in order to ensure the diagnosis of CHD. 

The TCM diagnosis of BSS will follow the principles described in “Criteria for TCM syndrome differentiation of patients with coronary heart disease,” published by the China Society of Integrated Traditional Chinese and Western Medicine [13].

### 2.3. Subject Inclusion and Exclusion

To participate in this study, subjects must be 35 to 75 years of age and must either have a previous history of myocardial infarction or have at least one coronary artery stenosis ≥50% confirmed by coronary angiography. In addition, subjects must currently be hospitalized with either unstable angina or an acute non-ST segment elevation myocardial infarction and have taken statin medicine for at least 1 month. From a TCM perspective, study participants must have the diagnosis of BSS. Finally, subjects must have a serum hsCRP level between 3 mg/L and 15 mg/L.

The criteria for exclusion include infection, fever, trauma, burn injury, or surgery within one month prior to recruitment; a concomitant diagnosis of cancer, sexually transmitted diseases, tuberculosis, or rheumatoid arthritis or other autoimmune diseases; or a history of serious pulmonary, hepatic, renal, neurological, psychiatric, or hematological diseases. In addition, subjects must not have previously undergone or currently be planning to undergo surgical intervention for CHD. Patients with severe heart failure indicated by an ejection fraction <35% will be excluded as will those with a reduced platelet count or a tendency to bleed or hemorrhage. Study participants must not currently be taking antibiotics or using TCM preparations that relieve fever or clear internal heat. Finally, patients who may become noncompliant or may participate in other clinical trials will be excluded.

During the course of the study, a subject may be excluded if (1) it is discovered that a subject was misdiagnosed for either BSS or CHD or was otherwise inappropriately accepted for participation in the trial; (2) if a subject misses a significant number of treatments or is missing significant records of data; (3) if subject's blood samples from the first two phases of the study (see Section 2.9) are contaminated or damaged in any way such that reliable data cannot be obtained; or (4) if for any reason (e.g., onset of a mid-study nosocomial infection), patient's inflammatory marker levels may not accurately reflect atherosclerotic inflammation. 

### 2.4. Sample Size Estimation

Because the data required to perform an *a priori* sample size calculation for this study is not available, we have adopted the sample size of a comparable trial for use in this pilot study. The comparable trial investigated the effects of TCM on unstable angina patients undergoing percutaneous coronary intervention and involved 60 subjects randomized into two treatment groups of 30 each [14]. For our pilot study, we will recruit 72 patients to begin the trial, assuming conservatively that 20% of the participants in each treatment group will ultimately not complete the study.

### 2.5. Randomization and Blinding

A member of the Good Clinical Practice (GCP) Clinical Center of Xiyuan Hospital who is independent of the study will use SAS 9.2 software to perform a block randomization, generating a sequence of 72 random numbers in 1 : 1 allocation between the two groups. In order to conceal the generation, the outcomes will be conducted by another member of GCP Clinical Center using a central randomization; once a patient meets all the criteria, a random number will then be delivered by telephone to the clinical researchers, unblinding only after data is collected for the first phase of the study (see Section 2.9). The number delivered by telephone will determine whether the subject receives the control or experimental therapy. 

Because the color of the STS solution given to the experimental group is unique and challenging to emulate, blinding will be difficult to achieve at the physician and patient levels. In order to minimize biases as much as possible, all other potential sources of information that may reveal treatment allocation to patients (e.g., contents of case report form, patient's random number) will be judiciously guarded. Additionally, the details of treatment allocation will not be disclosed to any patient until the study has concluded, and all study participants will be discouraged from discussing with one another their involvement in the trial. Finally, the clinical researchers will strictly abide by the study's random design and will interact with the patients in each group with as few differences as possible.

Blinding will be maintained at the level of outcome assessment. The individuals performing laboratory blood analyses, data management, and statistical analyses will be independent of the clinical component of the study and will not be provided with any information that may reveal treatment allocation details. 

### 2.6. Ethics

This trial has been approved by the local institutional ethics committee (Xiyuan Hospital, China Academy of Chinese Medical Sciences, Beijing, China; (2012XL022-2)) and is registered at the Chinese Clinical Trial Registry (ChiCTR-TRC-12002361). All aspects of our study will be conducted with adherence to the current version of the Declaration of Helsinki, the guidelines established by the International Conference on Harmonization of Good Clinical Practice, and the laws of China. Informed written consent will be required of all study participants, none of whom will be denied a standard, accepted therapy. 

### 2.7. Treatment

Each patient in the standard Western therapy (control) group will receive 20 mg simvastatin orally once per evening, in addition to any other oral or IV Western medications or TCM preparations (aside from those listed as exclusion criteria, above) that are deemed appropriate for a standard treatment of the patient's individual condition. These other medications will be prescribed by physicians who are not associated with the trial.

In addition to receiving the treatments described above for the control group, each patient in the East-West integrative therapy (experimental) group will also receive a daily intravenous dose of 80 mg STS (10 mg per ampoule, Jiangsu Carefree Pharmaceutical Co., Ltd., national drug approval number: H31022558), diluted with 250 mL 0.9% NaCl solution.

All study participants will receive treatment as indicated above for 14 consecutive days. 

### 2.8. Outcome Parameters

Serum hsCRP level will serve as the primary outcome parameter in this study. The levels of other inflammatory mediators, including IL-6, TNF*α*, VCAM-1, CD40 and soluble CD40 ligand (sCD40L), MCP-1, and MMP-9, as measured by enzyme-linked immunosorbent assay (ELISA), will constitute secondary outcome parameters. Additional secondary parameters will include safety and the extent of improvement in angina and BSS symptoms. 

To assess safety, patients will be asked to report any side effects or changes in feelings that they have noticed since the previous phase of data collection. In addition, the results of routine blood, urine, and stool tests, liver and kidney function tests, coagulation tests, and electrocardiogram tests will also be considered in the evaluation of safety. Finally, the clinical researchers will carefully monitor all patients for the potential onset of adverse events, the most severe of which may include arrhythmias, heart failure, recurrent myocardial infarction, myocardial rupture, cerebral infarction, cerebral hemorrhage, sudden cardiac death, and all-cause death. 

To assess improvement in angina symptoms, a scoring system will be applied based on the frequency, duration, and intensity of angina episodes [15, 16]. Similarly, a scoring system will also be used to evaluate BSS based on changes in signs and symptoms such as angina, a pulse of choppy or knotted nature, ecchymoses, dark purple tongue, lips, and gums, and expanded sublingual veins [17].

### 2.9. Data Collection Phases

Table 1 indicates the data to be collected at each phase of the study. At Phase I, before treatment for the trial is initiated, each patient's full medical history will be recorded, along with vital signs, symptoms of present illness, and all current medications. Tongue and pulse examination will be performed by experienced TCM physicians. Laboratory tests will include routine blood, urine, and stool tests, an electrocardiogram, an hsCRP test, and various other tests measuring cardiac markers (creatine kinase, CK-MB; troponin T, cTnT; troponin I, cTnI), liver and kidney function, blood lipid and glucose, and coagulation. A 10 mL blood sample will also be collected from each patient, centrifuged, and stored at −80°C for subsequent measurement of other inflammatory mediators (IL-6, TNF*α*, VCAM-1, CD40, sCD40L, MCP-1, and MMP-9) at the end of the study. Finally, the severity of each patient's angina and BSS symptoms will be quantitatively scored.

Phase II marks the end of the 14-day treatment period, at which time the medications specific to this study (i.e., simvastatin and STS) will be discontinued and the patients reassessed. Again, vital signs, symptoms of present illness, tongue and pulse exam results, and all current medications will be recorded. Angina and BSS scoring will be repeated, along with all Phase I laboratory tests aside from those measuring cardiac markers and blood lipid and glucose. As in Phase I, a blood sample from each patient will be collected and preserved for subsequent analysis of secondary inflammatory factors at the end of the study. A preliminary, nonstatistical evaluation of treatment safety and efficacy will also be performed by the data collectors.

Thirty days after simvastatin and STS are discontinued, Phase III data will be recorded, including level of hsCRP, angina and BSS scores, vital signs, symptoms of present illness, tongue and pulse exam results, and all current medications. As before, a blood sample for analysis of secondary inflammatory factors will be collected, and treatment safety and efficacy will again be preliminarily evaluated.

### 2.10. Data Management

The principal investigator of this study will collaborate with the director of the GCP Clinical Center of Xiyuan Hospital to schedule and coordinate this clinical trial. The GCP Clinical Center will be responsible for randomizing subjects into treatment groups, monitoring research progress, managing the data, and performing statistical analyses. All those involved in these aspects of the trial will remain blind to the details of treatment allocation.

All patient data will be recorded by trained clinical researchers using a standardized, preprinted, and paper case report form (CRF). Collection, transportation and preservation of all blood samples will be performed by trained staff using a standardized procedure. The Laboratory of Cardiovascular Diseases at Xiyuan Hospital will analyze all blood samples and report all results to the clinical researchers for documentation. At the conclusion of the study, CRFs will be delivered to the GCP Clinical Center and examined for completeness by an individual unassociated with the data collection process. If complete, the CRF will be closed to further revision in preparation for data entry.

At the GCP Clinical Center, a data manager uninvolved with subsequent statistical analysis for the study will be responsible for overseeing data entry. To ensure the reliability of the recorded data, two individuals under the data manager will each independently input a copy of the CRF data into a special database (EpiData 3.1 software, http://www.epidata.dk/). If any data in the CRF is unclear, the data manager will submit a clarification form to the principal investigator of the study, who will then issue an inquiry for the clinical researchers to resolve as soon as possible. The data manager will confirm the correct data according to the clinical researchers' response.

A third individual under the data manager will proofread the two independently completed database records to ensure that they are identical and accurately represent the data in the CRF. If the database records are not identical, the data in question will be confirmed from the original CRF. Once complete, the database records will be locked to further revision.

### 2.11. Statistical Analysis

Statistical analyses will be performed using SPSS 15.0 software (SPSS, Inc., Chicago, IL, USA). A Student's *t*-test or Wilcoxon rank-sum test was used, as appropriate, for the analyses of intergroup differences of measurement data; *χ* ^2^ test or Fisher's exact test if necessary was used for comparison of enumeration data. All tests were two tailed and a statistical probability of <0.05 was considered significant. 

## 3. Discussion

Despite recent advances in the treatment and prevention of cardiovascular diseases, CHD remains a significant threat to global health. The use of intensified lipid-lowering therapy involving statins and other Western medications has been proposed in an effort to reduce residual cardiovascular risk [18]. The multinational JUPITER trial, however, clearly demonstrated that patients with healthy LDL-C levels according to standard guidelines may still be at elevated risk due to inflammation associated with atherosclerosis, as measured by hsCRP [19]. Controlling inflammation may therefore be critically important in the treatment of CHD, and to this end, our study will explore the effect of an East-West integrative therapy involving STS and simvastatin on elevated levels of inflammatory markers in CHD patients.

The inflammatory process of atherosclerosis begins when excess LDL particles accumulate in the arterial intima and undergo oxidative modification [20, 21], resulting in recruitment of circulating monocytes via factors including MCP-1 and VCAM-1 [22–24]. In the vascular subendothelium, the monocytes differentiate into macrophages and then become foam cells after endocytosis of the oxidized LDL complexes [25]. Expansion of the inflammatory reaction occurs as these lipid-laden foam cells form the necrotic core of the developing atherosclerotic lesion and release proinflammatory cytokines such as TNF*α* that recruit additional monocytes, dendritic cells, mast cells, and T cells to the area [26]. The extensive crosstalk of cytokines induces vascular smooth muscle cells to produce IL-6, resulting in CRP release by the liver via the acute-phase response [27]. The activated smooth muscle cells also migrate into the arterial intima and lay down fibrous deposits [28], ultimately forming the atherosclerotic plaques that contribute to the development of CHD. As the disease progresses, CD40L-mediated stimulation of MMP-9 expression by vascular smooth muscle cells plays a major role in plaque destabilization [29], altogether increasing the risk of plaque rupture and the onset of complications such as ACS. 

Elevated levels of the inflammatory mediators mentioned above may serve as useful tools for gauging a patient's disease state and cardiovascular risk. CRP is widely regarded as the most useful biomarker for assessing atherosclerotic diseases, not only because of its ease and reliability of measurement, its wide and dynamic range of concentrations, and its remarkable stability but also because its degree of elevation in the blood correlates significantly with the level of risk for future adverse cardiovascular events associated with advanced atherosclerosis, such as myocardial infarction and ischemic stroke [27, 30]. Notably, CRP level is not related to the risk of developing venous thrombosis, a vascular condition typically independent of atherosclerosis [30]. Moreover, Liuzzo et al. found that although CRP levels are significantly increased in patients with unstable angina, which is usually caused by CHD due to atherosclerosis, the biomarker's concentration is not elevated in patients with variant angina, which is caused not by atherosclerosis but by vasospasms of the coronary arteries [31]. These observations altogether suggest that, in the context of cardiovascular disease, the extent of CRP elevation predicts the extent of atherosclerotic inflammation, thereby making CRP level a suitable primary outcome parameter for our study.

The levels of IL-6, TNF*α*, VCAM-1, CD40, sCD40L, MCP-1, and MMP-9 were selected as secondary outcome parameters in this trial, as these inflammatory markers are less effective indicators of atherosclerotic disease status compared to CRP. Tayebjee et al. found that levels of sCD40L and MMP-9 are significantly higher in stable CHD patients than in healthy controls but also that these markers may not strongly predict the severity of CHD [32]. In addition, although Biasucci et al. found that elevated IL-6 levels predict worse prognosis in patients with unstable angina, the authors were able to detect IL-6 in only 61% of their unstable angina group [33]. Nevertheless, the analysis of these inflammatory markers in addition to CRP provides a broader perspective of the inflammatory state of each patient, allowing us to examine and interpret the effect of STS-simvastatin integrative therapy on inflammation reduction with greater depth. 

It is possible that the biomarker concentrations measured in our study may reflect systemic inflammation due to a cause other than atherosclerosis. Indeed, CRP is an acute phase protein released by the liver as part of the body's immune response to nonspecific disturbances, including infection, autoimmune disorders, trauma or surgery, and cancer [34]. To diminish the effect of this possibility, we will carefully consider the past and present medical histories of all eligible patients before enrollment and exclude any patients in whom inflammatory markers may not accurately reflect the state of CHD. Additionally, we will also monitor all study participants during the course of the trial for changes that may confound interpretation of biomarker data, such as acquisition of a nosocomial infection. 

The mechanisms by which tanshinone IIA and statins inhibit the inflammatory process of atherosclerosis are diverse, numerous, and in many cases shared (reviewed in [8, 35]). Using STS and simvastatin in combination may yield an additive, supplementary effect not only in the reduction of internal inflammation but also in the improvement of external symptoms associated with CHD. In this trial, we will examine the therapeutic efficacy of a STS-simvastatin integrative therapy in CHD patients by quantitatively scoring each study participant's angina and BSS symptoms and examining for any changes over the course of the trial. 

There is a risk for negative interactions in any combination drug therapy, but integrative treatments involving STS appear to be relatively safe. In a systematic review of 25 randomized controlled trials, Qiu et al. found that integrative therapies involving STS for unstable angina may produce fewer side effects than the component Western therapies alone [36]. Side effects that were associated with the integrative therapies included flushing, dizziness, bruising, gum bleeding, blood in the sputum, and swelling at the STS injection site, but none were considered severe and none resulted in discontinuation of treatment. Another clinical study, which used STS and simvastatin in combination to treat peripheral vascular disease also found the integrative treatment to be safer than the statin therapy alone [10]. Our study will continue to monitor the short- and long-term side effects of STS-simvastatin integrative therapy and verify the safety observations reported by previous trials.

Several limitations affect the strength of our study design. Due to the unavailability of data needed to perform an *a priori* calculation, we determined the sample size for this study by adopting the number used in a comparable trial that we previously completed [14]. As a result, it is possible that our current study design may have insufficient power to reveal statistical significance at the level we desire. In addition, because the color of the STS solution makes double-blinding difficult, our study will be subjected to potential expectation biases from both clinical researchers and study participants. We will attempt to minimize these biases by maintaining blinding at the patient level to the fullest extent possible and by having clinical researchers interact with all subjects as similarly as possible. Full blinding will be maintained at the data analysis level. Finally, our project represents a single-center study and our results may not be wholly generalizable due to a possible selection bias. In the future, multicenter double-blinded, randomized controlled trials are necessary in order to faithfully establish the potential of STS-statin integrative therapy in treating CHD.

Nevertheless, the results of this trial are expected to clarify the potential of an East-West integrative therapy involving STS and simvastatin in reducing atherosclerotic inflammation in CHD patients. Our results will additionally confirm the safety and efficacy of this integrative therapy in treating CHD. Finally, the protocol of our study will also provide a methodological foundation upon which future clinical studies of integrative medicine may develop. 

## Trial Status

This trial has begun to recruit patients from November 2012, and there has been 10 volunteers up to February 2013.

## Figures and Tables

**Table 1:** Data Collection Phases and Scheme.

Data collection phase	Phase I: pretreatment 0 day	Phase II: posttreatment 14 days	Phase III: posttreatment 30 days
Inclusion/exclusion criteria	*√*		
Signed informed consent	*√*		
Patient interview			
General health	*√*		
Past medical history	*√*		
Signs and symptoms of present illness	*√*	*√*	*√*
Current medications	*√*	*√*	*√*
Physical exam			
Tongue and pulse examination	*√*	*√*	*√*
Heart rate and blood pressure	*√*	*√*	*√*
Laboratory tests			
Routine blood, urine, and stool (+ occult blood)	*√*	*√*	
Liver and kidney function	*√*	*√*	
Blood lipid and glucose	*√*		
Coagulation	*√*	*√*	
High-sensitivity CRP	*√*	*√*	*√*
Cardiac markers: CK-MB, cTnT, cTnI	*√*		
Electrocardiogram	*√*	*√*	
Inflammatory markers*	*√*	*√*	*√*
Assessment using TCM and Western medicine	*√*	*√*	*√*
Angina and BSS score	*√*	*√*	*√*
Randomization into treatment groups	*√*		
Overall evaluation			
Evaluation of efficacy		*√*	*√*
Evaluation of compliance		*√*	*√*
Evaluation of safety and side effects		*√*	*√*

*NOTE: blood sample will be stored for analysis at the end of the study. Inflammatory markers include interleukin-6 (IL-6), tumor necrosis factor alpha (TNF*α*), vascular cell adhesion molecule (VCAM-1), CD40 antigen and CD40 ligand (sCD40L), monocyte chemotactic protein-1 (MCP-1), and matrix metalloproteinase-9 (MMP-9).

## Abbreviations

ACS: Acute coronary syndrome
BSS: Blood stasis syndrome
sCD40L: Soluble CD40 ligand
CHD: Coronary heart disease
CK-MB: Creatine kinase
CRF: Case report form
CRP: C-reactive protein
hsCRP: High-sensitivity C-reactive protein
ELISA: Enzyme-linked immunosorbent assay
GCP: Good Clinical Practice
IL-6: Interleukin-6
LDL-C: Low-density lipoprotein cholesterol
MCP-1: Monocyte chemotactic protein-1
MMP-9: Matrix metalloproteinase-9
STS: Sodium tanshinone IIA sulfate
TCM: Traditional Chinese medicine
TNF*α*: Tumor necrosis factor alpha
cTnI: Troponin I
cTnT: Troponin T
VCAM-1: Vascular cell adhesion molecule-1.
